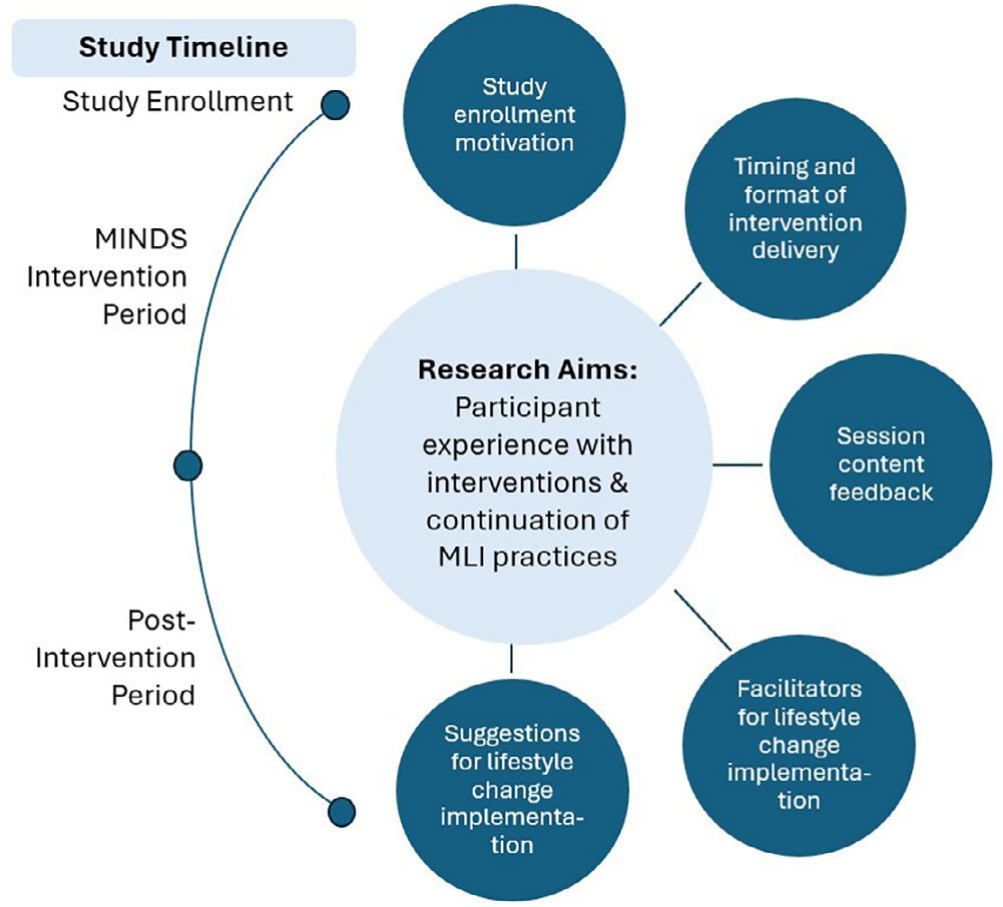# The Experience of Older Adults with Neurodegenrative Disease Risks with Virtual Multidisciplinary Lifestyle Interventions: A Qualitative Study

**DOI:** 10.1002/alz70861_108241

**Published:** 2025-12-23

**Authors:** Vineet Punia, Sara Taylor

**Affiliations:** ^1^ Cleveland Clinic, Cleveland, OH USA

## Abstract

**Background:**

The development of preventative and mitigating methods for neurodegenerative disorders, including dementia, among older adults is an urgent imperative. Emerging evidence suggests that multidisciplinary lifestyle interventions (MLI) could be effective and made more accessible if delivered virtually. We sought participant feedback on their experiences with a virtual MLI program and their plans for self‐guided continuation of the practices to gain insights into future virtual MLI programs for clinical care.

**Method:**

The Multidisciplinary lifestyle Interventions for Neurological Disorders during the Silent phase (MINDS) study is a randomized control trial for older adults (≥50 years) at risk of developing neurodegenerative disorders with the intervention arm receiving a 12‐week virtual MLI, including yoga, music therapy, cognitive training, and diet & nutrition education. We administered a post‐intervention survey to get participant feedback on their experiences with MLI. Participant responses were included based on availability at a cut‐off date for a sufficient range of responses and feasible in‐depth thematic analysis. We conducted descriptive statistics for finite responses and thematic analysis for open‐ended text responses.

**Result:**

We analyzed the responses from 22 participants [mean age 63 years (51 ‐ 75); mostly female (n = 18, 82%), white (n = 17, 77%), and non‐Hispanic (n = 17, 77%)]. We identified the following themes and sub‐themes: study enrollment motivation, timing and format of delivery (time commitment, format of delivery: virtual vs. in‐person trade‐offs), session content feedback, facilitators for lifestyle change implementation, and suggestions for lifestyle change implementation. Participants reported an overwhelmingly positive experience with the virtual MLI program. All participants indicated a high likelihood of continuing the lifestyle changes beyond the intervention period, with nutritional (86%) and cognitive training (64%) being most endorsed.

**Conclusion:**

Our findings suggest that virtual delivery of MLI to neurologically at‐risk older adults is feasible and effective in engaging its recipients. Incorporating participant‐driven feedback from our study can improve MLI‐based clinical care programs promoting brain health. Offering flexible scheduling, hybrid formats, and tailored resources could enhance recipients' engagement and adherence.